# Genomic Distinctions of LA-MRSA ST398 on Dairy Farms From Different German Federal States With a Low Risk of Severe Human Infections

**DOI:** 10.3389/fmicb.2020.575321

**Published:** 2021-01-08

**Authors:** Tobias Lienen, Arne Schnitt, Jens Andre Hammerl, Sven Maurischat, Bernd-Alois Tenhagen

**Affiliations:** Department of Biological Safety, German Federal Institute for Risk Assessment, Berlin, Germany

**Keywords:** LA-MRSA, dairy farms, phylogenetic relationship, antimicrobial resistance, one health

## Abstract

Methicillin-resistant *Staphylococcus aureus* (MRSA) have been found on German dairy farms and may be the cause of difficult-to-treat bovine mastitis. Considering the one health approach, MRSA might be transmitted from animals to humans raising the risk for severe infections. On 17 German dairy farms with a history of MRSA detection, MRSA strains were isolated from quarter milk, bulk tank milk, and swab samples of calves, heifers, pigs, and the environment. A selection of 33 isolates was analyzed using whole-genome sequencing and antimicrobial resistance testing. All detected MRSA strains were attributed to the livestock-associated sequence type 398. Methicillin-resistance was associated with the *mecA* gene in the staphylococcal cassette chromosome (SCC)*mec* types IVa (7/33) or V (26/33). The MRSA strains across the German federal states showed large allelic differences indicating independent development and distribution. On one farm, a clonal MRSA isolate was widely spread among different animals and the milking equipment. Moreover, MRSA transmission between two dairy farms in one federal state seems to be likely. In depth studies indicated that the resistance gene prediction and phenotypic resistance are in good agreement. Twenty eight strains were determined to exhibit a non-wildtype phenotype (resistant) against up to seven antimicrobial substances with an overall resistance to β-lactams and tetracycline. Ten different phenotypic antimicrobial resistance patterns were found among the MRSA strains. The strains harbored a wide virulence gene repertoire, of which some of them are related to bovine mastitis. However, the isolates lacked typical human infection associated factors such as the immune evasion cluster genes, staphylococcal enterotoxin genes, or Panton-Valentine leukocidin genes leading to the assumption for a low risk for severe human infections and foodborne diseases.

## Introduction

Methicillin-resistant *Staphylococcus* (*S*.) *aureus* (MRSA) were repeatedly detected on German dairy farms (Tenhagen et al., 2018; Kadlec et al., 2019) and may be a cause of bovine mastitis (Holmes and Zadoks, 2011). MRSA infections are hard to cure since these bacteria are resistant against β-lactam antibiotics, which are widely used for *S. aureus* mastitis treatment. In addition to the animal health aspect, MRSA may be transmitted from animals to the farm personnel and sporadically cause severe infections in humans such as dermatitis, otitis, wound infection, pneumonia, endocarditis, or sepsis (Goerge et al., 2017). MRSA may carry resistance genes against several classes of antibiotics and even resistance against last resort antibiotics such as linezolid was found in isolates from various livestock (Cuny et al., 2017). Moreover, MRSA can be equipped with a wide arsenal of virulence factors such as immune evasion clusters (IECs), toxins, or leukocidins. Both, antibiotic resistance and virulence genes, are often encoded on mobile genetic elements (MGEs) giving the possibility to spread resistance or virulence between different strains. The most common MGE in MRSA with regard to antibiotic resistance is the staphylococcal cassette chromosome (SCC)*mec* element, in which the β-lactam antibiotic resistance gene *mecA* or its homolog *mecC* is located. The SCC*mec* is structurally divided into the types I–XIII (Lakhundi and Zhang, 2018). Moreover, antimicrobial resistance determinants may also be encoded on plasmids (Feßler et al., 2018). Likewise, also virulence factors are found in MGEs across the MRSA genome, e.g., in *S. aureus* pathogenicity islands (SaPIs) or phages. Livestock-associated MRSA (LA-MRSA) often lack the potential for causing severe human infections due to a lack of IEC genes or genes encoding the toxic shock syndrome toxin (TSST) or Panton-Valentine leucocidin (PVL; Cuny et al., 2015a). However, frequent monitoring of LA-MRSA strains from different livestock farms and the respective environment are necessary, since the genetic repertoire of MRSA strains might change spontaneously due to horizontal gene transfer leading to more harming strains with regard to animal and human health (Kraushaar et al., 2017).

The aim of the study was to compare the genotypes, antimicrobial resistance profiles, and virulence factors of MRSA strains from 17 dairy farms in eight German federal states. Whole-genome sequencing (WGS) of selected strains was conducted and the sequence data were analyzed regarding antimicrobial resistance genes and virulence factors to draw conclusions for a potential public health risk. Furthermore, the phylogenetic relationship between MRSA strains from various regions as well as within one farm was analyzed by core genome multi-locus sequence typing (cgMLST).

## Materials and Methods

### Sampling and MRSA Strain Selection

For this study, 17 dairy farms across eight German federal states were selected due to a previous positive MRSA detection. Samples from bovine mammary quarters (quarter milk samples, QMS), bulk tank milk (BTM), calves (nasal swabs), heifers (nasal swabs and udder cleft swabs), pigs (nasal swabs) on dairy farms as well as the milking equipment and environment, which were retrieved in a sampling campaign from September 2018 to December 2019, were examined for MRSA. Milk (1 ml) and swab samples were examined using a double selective enrichment method by incubation in Mueller Hinton broth (Thermo Fisher Scientific Oxoid Ltd., United Kingdom) supplemented with 6% of NaCl, tryptic soy broth (Merck, Germany) supplemented with 3.5 mg/l cefoxitin (Sigma-Aldrich, United States) and 50 mg/l aztreonam (Sigma-Aldrich, United States) and subsequent incubation on mannitol salt (Thermo Fisher Scientific Oxoid Ltd., United Kingdom) agar plates containing 4 mg/l cefoxitin (Sigma-Aldrich, United States). Each incubation step lasted for 24 ± 2 h at 37°C. With regard to a potential food intoxication or transmission to humans by the consumption of MRSA contaminated milk, in particular strains from QMS and BTM were chosen for sequencing if available. In total, 33 out of 184 MRSA isolates were selected as most interesting for comparison by WGS according to previous PCR results with regard to SCC*mec* type and *spa* type (Schnitt et al., 2020). In Table 1, all sequenced MRSA strains are listed. The strains originated from QMS, BTM, nasal swabs of calves, heifers, and a pig as well as a swab from a teatcup and a teat cleaning water sample. The data were anonymized due to the general data protection regulation. The code is a combination of the German federal state, the farm in the respective federal state and the sample number of the respective farm, e.g., AA1 means German federal state A, farm A from this federal state and sample number 1 from this farm. For studying the transmission of MRSA strains across one farm, isolated strains from various sample types were included from farm AA.

**TABLE 1:** Overview of sequenced MRSA strains, source, SCC*mec*-, *spa*-type, ST, prediction of antimicrobial resistance genes, and phenotypic resistance.

Nr	Code	Source	SCC*mec*-type	*spa*-type	ST^5^	Predicted antimicrobial resistance genes	Phenotypic resistance^6^
1	AA1	QMS^1^	IVa	t011	398	*aac(6’)Ie-aph(2”)Ia;blaZ;dfrK;mecA;str;tet*(M)	FOX, GEN, KAN, PEN, STR, TET, TMP
2	AA2	QMS	IVa	t011	398	*aac(6’)Ie-aph(2”)Ia;blaZ;dfrK;mecA;str;tet*(M)	FOX, GEN, KAN, PEN, STR, TET, TMP
3	AA3	QMS	IVa	t011	398	*aac(6’)Ie-aph(2”)Ia;blaZ;dfrK;mecA;str;tet*(M)	FOX, GEN, KAN, PEN, STR, TET, TMP
4	AA4	Pig	V	t1451	398	*blaZ;erm*(A)*;mecA;tet*(M)*;spc;vga*(E)	FOX, ERY, PEN, TET, TIA
5	AA5	Calf	IVa	t011	398	*aac(6’)Ie-aph(2”)Ia;blaZ;dfrK;mecA;tet(M)*	FOX, GEN, KAN, PEN, STR, TET, TMP
6	AA6	Heifer	V	t1451	398	*blaZ;erm*(A)*;mecA;tet*(M)*;spc;vga*(E)	FOX, ERY, PEN, TET, TIA
7	AA7	TC^2^	IVa	t011	398	*aac(6’)Ie-aph(2”)Ia;blaZ;dfrK;mecA;str;tet*(M)	FOX, GEN, KAN, PEN, STR, TET, TMP
8	AA8	BTM^3^	IVa	t011	398	*aac(6’)Ie-aph(2”)Ia;blaZ;dfrK;mecA;str;tet*(M)	FOX, GEN, KAN, PEN, STR, TET, TMP
9	AA9	TCW^4^	IVa	t011	398	*aac(6’)Ie-aph(2”)Ia;blaZ;dfrK;mecA;str;tet*(M)	FOX, GEN, KAN, PEN, STR, TET, TMP
10	BA1	QMS	V	t011	398	*blaZ;mecA;str;tet*(K)*;tet*(M)*;vga*(A)	FOX, PEN, STR, TET, TIA
11	BB1	QMS	V	t034	398	*dfrG;erm*(A)*;mecA;tet*(K)*;tet*(M)*;spc;vga*(E)	FOX, CLI, ERY, PEN, TET, TIA, TMP
12	BC1	QMS	V	t011	398	*blaZ;mecA;tet*(K)*;tet*(M)	FOX, PEN, TET
13	BC2	BTM	V	t011	398	*blaZ;mecA;tet*(K)*;tet*(M)	FOX, PEN, TET
14	CA1	QMS	V	t034	398	*dfrG;lnu*(B)*;lsa*(E)*;mecA;tet*(K)*;tet*(M)*; spc*	FOX, CLI, PEN, Q–D, TET, TIA, TMP
15	CB1	QMS	V	t011	398	*blaZ;mecA;tet*(K)*;tet*(M)	FOX, PEN, TET
16	DA1	Calf	V	t011	398	*blaZ;mecA;tet*(K)*;tet*(M)	FOX, PEN, TET
17	DB1	Calf	V	t571	398	*dfrG;erm*(A)*;mecA;tet*(K)*;tet*(M)*;spc;vga*(E)	FOX, CLI, ERY, PEN, TET, TIA, TMP
18	EA1	BTM	V	t011	398	*blaZ;mecA;tet*(K)*;tet*(M)*;vga*(A)	FOX, PEN, TET, TIA
19	EB1	QMS	V	t034	398	*blaZ;dfrG;mecA;tet*(K)*;tet*(M)*;vga*(A)	FOX, PEN, TET, TIA, TMP
20	EB2	QMS	V	t034	398	*blaZ;dfrG;mecA;tet*(K)*;tet*(M)*;vga*(A)	FOX, PEN, TET, TIA, TMP
21	EC1	QMS	V	t034	398	*blaZ;dfrG;mecA;tet(M);vga*(A)	FOX, CLI, PEN, TET, TIA, TMP
22	EC2	QMS	V	t011	398	*blaZ;dfrG;mecA;tet*(K)*;tet*(M)*;vga*(A)	FOX, PEN, TET, [TIA], TMP
23	ED1	Heifer	V	t034	398	*blaZ;dfrG;mecA;tet*(K)*;tet*(M)*;vga*(A)	FOX, PEN, TET, TIA, TMP
24	ED2	BTM	V	t1928	398	*blaZ;dfrG;mecA;tet*(K)*;tet*(M)*;vga*(A)	FOX, PEN, TET, TIA, TMP
25	EE1	QMS	V	t011	398	*blaZ;dfrG;mecA;tet*(K)*;tet*(M)*;vga*(A)	FOX, PEN, TET, [TIA], TMP
26	EE2	QMS	V	t011	398	*blaZ;dfrG;mecA;tet*(K)*;tet*(M)*;vga*(A)	FOX, PEN, TET, TIA, TMP
27	EE3	QMS	V	t011	398	*blaZ;dfrG;mecA;tet*(K)*;tet*(M)*;vga*(A)	FOX, PEN, TET, TIA, TMP
28	EE4	QMS	V	t1928	398	*blaZ;dfrG;mecA;tet*(K)*;tet*(M)*;vga*(A)	FOX, PEN, TET, TIA, TMP
29	EE5	QMS	V	t1928	398	*blaZ;dfrG;mecA;tet*(K)*;tet*(M)*;vga*(A)	FOX, PEN, TET, [TIA], TMP
30	FA1	Calf	V	t034	398	*blaZ;dfrG;fexA;mecA;tet*(K)*;tet*(M)	FOX, CHL, PEN, TET, TMP
31	FB1	QMS	V	t2011	398	*blaZ;mecA;tet*(K)*;tet*(M)	FOX, PEN, TET
32	GA1	BTM	V	t034	398	*dfrG;erm*(A)*;mecA;tet*(K)*;spc;vga*(E)	FOX, CLI, ERY, PEN, TET, TIA, TMP
33	HA1	Calf	V	t034	398	*blaZ;dfrG;mecA;tet*(K)*;tet*(M)	FOX, PEN, TET, TMP

*Underlined resistances were not predicted. Resistances in brackets were predicted but not detected phenotypically. ^1^QMS, Quarter milk sample. ^2^TC, Teat cup. ^3^BTM, Bulk tank milk. ^4^TCW, Teat cleaning water. ^5^ST, Sequence type. ^6^FOX, Cefoxitin; GEN, Gentamycin; KAN, Kanamycin; PEN, Penicillin; STR, Streptomycin; TET, Tetracycline; TMP, Trimethoprim; CLI, Clindamycin; ERY, Erythromycin; TIA, Tiamulin; Q–D, Quinupristin–Dalfopristin; CHL, Chloramphenicol.*

### DNA Extraction and WGS

Methicillin-resistant *Staphylococcus aureus* isolates were cultured on sheep blood agar (Oxoid GmbH, 46483, Wesel, Germany) and DNA of one inoculation loop filled with MRSA colonies was extracted using the Qiagen DNeasy Blood and Tissue Kit (Qiagen, Germany) according to the manufacturer’s protocol modified by adding 10 μl lysostaphin to the lysis buffer. The DNA library was prepared using an Illumina Nextera DNA Flex kit (Illumina Inc., United States) and the 150 bp paired-end sequencing run was performed on an Illumina NextSeq 500 instrument.

### Bioinformatic Analyses

#### Assembly and Quality Control

Raw Illumina reads were trimmed and *de novo* assembled with the in-house developed Aquamis pipeline^1^ which implements fastp (Chen et al., 2018) for trimming and shovill (based on SPAdes)^2^ for assembly. Furthermore, it performs mash v 2.1 for reference search (Ondov et al., 2016) as well as quast v 5.0.2 for assembly quality control (Mikheenko et al., 2018). The minimal coverage depth was >80. Quality of assemblies was checked by single-copy and duplicated orthologs analyses. The fraction majority species was >0.97. The total genome length was >2.7 Mbp.

### Phylogenetic Analyses

The MLST sequence type was inferred using mlst^3^ with the pubmlst database (Jolley and Maiden, 2010). Moreover, SCC*mec*- and *spa*-types were predicted with respect to the software tools SCCmecFinder 1.2 and spaTyper 1.0 of the Centre for Genomic Epidemiology.^4^ In addition, the phylogenetic relationship of all sequenced MRSA strains was analyzed using cgMLST in Ridom SeqSphere + version 7.0.4. The default settings were kept so that clusters were defined at less than 24 allelic differences.

### AMR Genes

Bacterial characterization was conducted with the in-house developed Bakcharak pipeline,^5^ which implements ABRicate^6^ for screening of antimicrobial resistance genes using the NCBI amrfinder database (Feldgarden et al., 2019).

### Virulence Factor Genes

Virulence factor genes were predicted using the VFDB (Chen et al., 2005). Following the staphylococcal virulence factor classification of Naushad et al. (2019), the detected virulence factor genes were attributed to the functional categories adhesion, exoenzymes, hemolysis, immune evasion, iron uptake, and metabolism or secretion. A detailed sequence search for SaPIs and phages in the obtained sequences was performed using the NCBI blastn suite. Therefore, a collection of SaPIs and phages (phiNM3, phi80, phiPVL, phiETA, Saeq1, SaPI1-3, SaPIbov1-2, SaPIbov4-5, SaPIeq1, SaPIivm10, SaPIishikawa11, SaPIivm60, SaPIino10, SaPIhirosaki4, SaPIj11, SaPIhhms2, SaPINN54, SaPIPM1, SaPI68111, and SaPIj50) was chosen according to the publications of Walther et al. (2018) and Alibayov et al. (2014).

### Antimicrobial Susceptibility Testing

Antimicrobial susceptibility testing (AST) was performed by broth microdilution according to the CLSI standard (ISO 20776-1:2006 or CLSI M31-A3) using a standardized antibiotic panel (EUVENC scheme) that is used in all member states of the European Union for resistance monitoring on staphylococci from livestock and food. For evaluation of minimal inhibitory concentrations (MIC) of the individual isolates the clinical breakpoints values of the CLSI were used. For quality control of resistance testing the *S. aureus* isolates ATCC 29213 and ATCC 25923 were used.

## Results

### MLST-, SCC*mec*-, and *spa*-Typing of MRSA Strains

Analyses of the sequence data showed that all strains belonged to the sequence type (ST) 398 (Table 1). MRSA strains with SCC*mec* type V dominated on the dairy farms. Solely on one farm AA, MRSA strains with SCC*mec* type IVa were detected in BTM, QMS, a nasal swab of a calf, a teatcup, and the teat cleaning water, whereas the MRSA strains AA4 (pig) and AA6 (heifer) from the animals placed in the pig barn carried SCC*mec* type V. Regarding the *spa*-types, t011, and t034 were mostly found. Beside, also *spa*-types t1451, t571, t1928, and t2011 were detected. On four farms (AA, EC, ED, and EE) various *spa*-types were found in different sample types. On farm AA and in accordance with the varying SCC*mec* types, *spa*-type t011 was found in BTM, QMS, the nasal swab of a calf, the teatcup and the teat cleaning water, whereas the MRSA strains from the nasal swabs of a pig and a heifer located in the pig barn carried *spa*-type t1451. On farm EC, two different *spa*-types (t011 and t034) were found in QMS. The *spa*-types t034 (nasal swab of heifer) and t1928 (BTM) were detected on farm ED. Moreover, on farm EE, different *spa*-types (t011 and t1928) were found in QMS.

### Antimicrobial Resistance Profiles

A broad range of antimicrobial resistance genes was detected in the sequences of the different MRSA strains. Resistance to the antibiotic classes aminoglycoside, β-lactam, trimethoprim, tetracycline, macrolide, streptogramin, lincosamide, and phenicol were predicted showing differences between the dairy farms and sample types on farm AA (Table 1). All MRSA strains (33/33) carried the *mecA* gene, whereas the β-lactamase encoding *blaZ* gene was missing in four strains. Resistance to the other antibiotic classes was encoded by the following genes; aminoglycoside [*aac(6’)Ie-aph(2”)Ia*, *str*], aminocyclitol (*spc*), macrolide-lincosamide-streptogramin B [*erm*(A)], trimethoprim (*dfrG, dfrK*), tetracycline [*tet*(K), *tet*(M)], pleuromutilin–lincosamide–streptogramin A [*lsa*(E), *vga*(A), *vga*(E)], lincosamides [*lnu*(B)], and phenicol (*fexA*). All MRSA strains (33/33) harbored tetracycline resistance genes. Resistance to aminoglycosides (14/33), trimethoprim (24/33), and pleuromutilin–lincosamide–streptogramin A (19/33) was also commonly predicted. In contrast, resistance to macrolides–lincosamide–streptogramin B (5/33) and phenicol (1/33) was less frequently predicted.

The phenotypic resistance according to the MIC values was in good agreement with the predicted antimicrobial resistance genes (Table 1). The genotypic differences between the strains throughout the farms were also shown in the phenotypic resistance pattern. All MRSA strains were resistant to cefoxitin, penicillin, and tetracycline. Resistance to trimethoprim and tiamulin was also widespread. Only a few strains showed resistance to gentamicin (7/33), kanamycin (7/33), streptomycin (8/33), clindamycin (5/33), or erythromycin (5/33). Only the strain CA1 was phenotypically resistant to quinupristin–dalfopristin and strain FA1 was phenotypically resistant to chloramphenicol. In sum, ten different phenotypic resistance patterns occurred within the various MRSA strains.

### Virulence Factors

The MRSA strains showed a diverse repertoire of virulence factor genes. Most of the analyzed genes (45/63) were present in every MRSA strain (Table 2). The *cna* (6/33), *sdrE* (32/33), and von Willebrand factor binding protein (32/33) genes were not found in all strains. Typical human MRSA IEC genes such as *scn*, *sak*, and *chp* or exfoliative toxin genes (*eta*/*etb*) were not detected in any of the sequenced isolates. Likewise, genes encoding toxins such as staphylococcal enterotoxins (SE; *sea, seb, sec, sed, see, seh, selk, sell, selq*) or TSST (*tsst*) were not found. Regarding PVL, the leukocidin subunit *lukF-PV* gene was only detected in seven MRSA strains, whereas the *lukS-PV* gene, which encodes the other PVL subunit, was not found. The pathogenicity island SaPIbov5 was detected in eight MRSA strains. Seven of these were isolates from the same farm AA. SaPIbov4 was found in eleven strains; with a sequence coverage regarding the reference of 89–90%. Other SaPIs or phages were not detected in the assembled sequences.

**TABLE 2:** Number of MRSA strains harboring various predicted virulence associated genes and corresponding functional categories.

Function	Predicted virulence factor genes	No. of strains
Adhesion	*cap, clfA/B, ebp, fnbA, map, sdrC/D*	33 (100%)
	*can*	6 (18%)
	*sdrE*	32 (97%)
Biofilm formation	*icaA/B/C/D/R*	33 (100%)
Exoenzymes	*aur, geh, lip, hysA, sspA/B/C*	33 (100%)
	von Willebrand factor binding protein	32 (97%)
Hemolysis	*hla, hlb, hld, hlgA/B/C*	33 (100%)
Immune evasion	*coa, spa, sbi*	33 (100%)
Iron uptake and metabolism	*isdA/B/C/D/E/F/G, srtB*	33 (100%)
Secretion	*esaA/B/C, essA/B/C, esxA/B*	33 (100%)
Toxin	*lukF-PV*	7 (21%)
	*lukS-PV*	0
	*tsst*	0
	*sea, seb, sec, sed, see, seh, selk, sell, selq*	0
IEC^1^	*chp, scn, sak*	0
MGE^2^	SaPIbov4	11 (33%)
	SaPIbov5	8 (24%)

*^1^IEC, Immune evasion cluster. ^2^MGE, Mobile genetic element.*

### Phylogenetic Relationship

**FIGURE 1:**
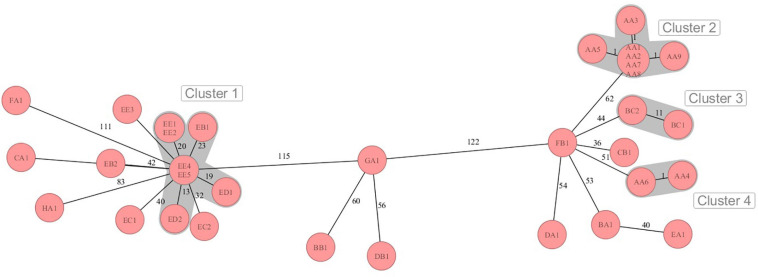
Minimum spanning tree visualization of cgMLST analysis of the MRSA strains on dairy farms from eight German federal states A-H with four clusters of close phylogenetic relationship. Numbers represent the allelic differences between the MRSA strains.

The sequences were analyzed by cgMLST regarding the phylogenetic relationship of the MRSA strains from the dairy farms across the eight German federal states. Four different clusters were retrieved in the minimum spanning tree (MST) analysis (Figure 1). The MST analysis showed large allelic differences between the MRSA strains from different German federal states and also between farms from the same region. Moreover, MRSA strains from the same farm (EB, EC, and EE) sometimes differed significantly in the core genome. The MRSA strains EB1, ED1/2, and EE1/2/4/5 from three farms from German federal state E (cluster 2) clustered with 13–23 alleles differences closer together in comparison to the farms from other federal states. On farm AA, MRSA strains of the sample types QMS (AA1–3), BTM (AA8), the nasal swab of a calf (AA5) as well as the samples of the teatcup (AA7) and teat cleaning water (AA9) clustered closely together with a maximum difference of one core genome allele. Contrary to this, the strains AA4 (nasal swab of pig) and AA6 (nasal swab of heifer), with a pig barn origin, formed an own cluster, which is in accordance with the different SCC*mec*- and *spa*-types.

## Discussion

Methicillin-resistant *Staphylococcus aureus* may be widespread on German dairy farms potentially causing infections such as bovine mastitis (Schnitt and Tenhagen, 2019). In addition to animal health and with regard to a transmission from animals to humans, monitoring of MRSA abundance and genotyping is also important in the public health context.

### Genomic Distinctions Between MRSA Isolates Across Germany

In our study, 33 MRSA strains from 17 dairy farms across eight German federal states were phenotypically and genotypically analyzed. All strains were characterized as ST398 LA-MRSA, a sequence type, which is often found on dairy farms (Feßler et al., 2012; Kadlec et al., 2019) and which is the most widely disseminated LA-MRSA sequence type (Cuny et al., 2015b). SCC*mec*-types IV and V as well as the *spa*-type t011 dominated in LA-MRSA on Dutch dairy farms as reported by Feßler et al. (2012). This is in accordance with the results of our study, however, in contrast, SCC*mec*-type IVa was only found in 1/17 farms and the *spa*-type t034 co-dominated to the *spa*-type t011. Tenhagen et al. (2018) also detected the *spa*-types t011 and t034 as most frequent in MRSA of BTM from German dairy farms. Moreover, a dominance of MRSA carrying SCC*mec* type V on German dairy farms was shown in the study of Kadlec et al. (2019).

The phylogenetic analysis of the MRSA strains done by cgMLST showed mainly distinct allelic differences and only a few clusters of close relationship. Although the strains were similar according to SCC*mec*- or *spa*-typing, the core genome differed in some cases by more than 120 alleles. This illustrates the high genomic plasticity and strong evolution in terms of genetic recombination in MRSA. Although exhibiting differences of 56–60 alleles, the strains GA1, BB1, and DB1 clustered together in the MST. The phylogenetic relation of these strains was also indicated by the similar antimicrobial resistance genes profile and the identical phenotypic resistance pattern. Only some MRSA strains from federal state E showed lower genetic divergence, in particular the strains ED2 and EE4/5. These strains also shared the same *spa*-type t1928. It can be speculated that a transmission of these MRSA strains between the farms took place. Reasons for this might be humans, e.g., farm personnel or veterinarians, or animal trade transmitting MRSA from one farm to another. In line with that, the introduction and transmission of MRSA on pig farms in Norway by farm workers or livestock trade was illustrated in the studies of Grontvedt et al. (2016) and Elstrøm et al. (2019). The potential role of “humans” as MRSA vectors across dairy farms was also shown in a recently published review of Schnitt and Tenhagen (2019).

Since the MRSA genotype might also vary within farms, strains of different sample types from selected farms were investigated. MRSA strains with a maximum of one allele difference in cgMLST were found in BTM, QMS of different cows, a nasal swab of a calf, the teatcup of the milking equipment and water for teat cleaning prior to milking on farm AA. Moreover, the strains harbored the same antimicrobial resistance genes and showed the same phenotypic resistance pattern. Therefore, the spread of one clonal LA-MRSA strain between cows, calves, and the milking equipment seems likely. In contrast, the MRSA isolates of the pig and heifer placed in the pig barn showed a MRSA strain, which differed genotypically from the strain in the dairy barn. A spillover of MRSA from the pig production to veal calves or dairy cows was suggested by several studies (Locatelli et al., 2017; Hansen et al., 2019). However, in our study, the spillover from pigs to other animals was only found in close proximity in the pig barn. Various MRSA genotypes also co-existed on farms EB, EC, and EE. Accordingly, Feßler et al. (2012) showed a co-dominance of several MRSA strains on one dairy farm. A reason for this might be the purchase of new animals and thus MRSA genotypes in the farm or transmission of additional strains by humans or other vectors. On the other side, genetic diversification might have appeared on the farm leading to altered MRSA genotypes.

### Widespread Multi-Resistant MRSA

The MRSA strains in our study carried a broad repertoire of antimicrobial resistance genes and 28 strains were multi-resistant to at least three classes of antibiotics. This is contrary to the recently published study of Kadlec et al. (2019), in which less than half of the MRSA strains from German dairy farms were multi-resistant with the constraint that only ten MRSA isolates were investigated by the authors. The prediction of the antimicrobial resistance genes in our study was mostly in agreement with the phenotypic antibiotic class resistance pattern. Resistance to β-lactam antibiotics such as cefoxitin or penicillin was mediated by the *mecA* gene in all strains, whereas the variant *mecC* gene was not found. In contrast, Schlotter et al. (2014) found MRSA harboring the *mecC* gene in 16 of 56 milk samples in a German dairy herd, probably due to a spread of this strain within the farm. However, in accordance with our study, MRSA strains in dairy cattle from Germany and Greece only harbored the *mecA* gene (Kadlec et al., 2019; Papadopoulos et al., 2019). The resistance to several classes of antibiotics was mediated by two to three different genes in our study. Feßler et al. (2018) summarized in their review that small plasmids play a pivotal role in the dissemination of certain antimicrobial resistance genes. Although not analyzed in detail, the transmission of resistance genes such as *vga*(A) or *dfrK* through plasmids likely also played a role in the strains of our study. In general, the antimicrobial resistance patterns differed between the farms indicating an independent development of the strains across Germany. Resistance to macrolides was rare in the MRSA strains. Accordingly, macrolide resistance in *S. aureus* retrieved from bovine mastitis was reported to be low (Pyorala et al., 2014; El Garch et al., 2020). In contrast, in a study of Tenhagen et al. (2018) macrolide resistance was detected in 17/41 MRSA isolates of BTM from German dairy farms. In our study, resistance to chloramphenicol was only found in one MRSA strain harboring the *fexA* gene. Accordingly, the *fexA* gene was only rarely found in a study analyzing ST398 MRSA isolates from bovine mastitis (Feßler et al., 2010). Resistance to tetracyclines was found in every strain from all farms in our study mediated by the *tet*(K) or *tet*(M) genes. In agreement with this, in the studies of Feßler et al. (2012) and Kadlec et al. (2019) all MRSA strains from dairy farms were resistant to tetracycline. Moreover, tetracycline resistance was detected in 99.4% of the MRSA isolates received from the cattle food chain (Tenhagen et al., 2014) and in 95.1% of the MRSA in BTM from German dairy farms (Tenhagen et al., 2018). Tetracyclines have been extensively used on animal farms, thus promoting the survival of tetracycline resistant strains (Granados-Chinchilla and Rodriguez, 2017). Furthermore, resistance to trimethoprim and tiamulin, a pleuromutilin, was detected in more than half of the MRSA strains in our study. Staphylococci are non-target bacteria with respect to pleuromutilins, however, their use in especially pig farming selects for multi-resistant MRSA (van Duijkeren et al., 2014). In this study, pleuromutilin resistance was transmitted by the *vga*(A) or *vga*(E) genes. In particular in the German federal state E, the *vga*(A) gene was present in MRSA strains from several farms. The *vga*(A) gene was shown to be transferred by plasmids (Feßler et al., 2018), whereas the *vga*(E) gene is located on a transferable transposon (Schwendener and Perreten, 2011). Hauschild et al. (2012) originally detected the *vga*(E) gene in dairy cattle. Although the *vga*(A) gene was reported to be most widespread among the *vga* genes (Feßler et al., 2018), in our study, also the *vga*(E) gene was equally distributed across the MRSA strains from the dairy farms. The resistance to trimethoprim was mediated by the *dfrG* or *dfrK* genes. The *dfrK* gene was only detected on farm AA in the MRSA strains with SCC*mec* type IVa. This is contrary to the finding of a 85.7% dissemination of the *dfrK* gene in MRSA isolates of bovine mastitis (Feßler et al., 2010). Moreover, the physical linkage of the *dfrK* and *tet*(L) genes, as described in Kadlec et al. (2012), was not found in our study. Aminoglycosides are widely used in veterinary medicine (EMA, 2018). Only the SCC*mec* IVa MRSA strains of farm AA showed phenotypical aminoglycoside resistance to streptomycin, gentamicin, and kanamycin. This can be explained by the different repertoire of antimicrobial resistance genes to aminoglycosides in the respective strains encoded by the *aac(6’)Ie-aph(2”)Ia*, and *str* genes. Moreover, the *spc* gene was detected in five strains, which mediates resistance to spectinomycin, an aminocyclitol, only (Zarate et al., 2018). The MRSA strain CA1 was phenotypically resistant to the streptogramin A and B quinupristin–dalfopristin. This is in agreement with the detection of the *lsa*(E) gene, since an eight-fold increased quinupristin–dalfopristin MIC was also previously detected for *S. aureus* harboring the *lsa*(E) gene (Wendlandt et al., 2013). Moreover, also resistance to clindamycin as detected for strain CA1 is associated with the *lsa*(E) gene (Wendlandt et al., 2013). Furthermore, *erm* genes confer inducible or constitutive resistance to macrolides, lincosamides, and streptogramin B (Leclercq, 2002). Therefore, in our study, the strains BB1 and GA1, which harbored the resistance gene *erm*(A), on the one hand showed phenotypical resistance to the macrolide erythromycin, but on the other side these strains were also resistant to the lincosamide clindamycin.

### Large Repertoire of Virulence Factor Genes

The LA-MRSA strains from the dairy farms in our study harbored multiple virulence associated genes, most of them present in all strains. Piccinini et al. (2010) postulated that a specific virulence gene combination is related to the development of subclinical mastitis and the prevalence of *S. aureus* in dairy herds. Moreover, Magro et al. (2017) related some of the virulence factors to more contagious *S. aureus* strains with regard to mastitis. In accordance with the prediction of the *hlb* gene, β-hemolysis on sheep blood agar was found for every detected MRSA strain in our study (data not shown). The presence of hemolysins as a factor for bovine mastitis in Russian dairy herds was described in the study of Fursova et al. (2020). In our study, the clumping factor encoding genes *clfA* and *clfB* were detected in all strains. This is in contrast to the study of virulence factor genes in dairy cattle from Brazil conducted by Klein et al. (2012), in which the prevalence of the *clfB* gene was higher (91.8%) than the *clfA* gene prevalence (50.6%). In particular, the ClfB protein is associated with *S. aureus* nasal colonization and skin infections in humans (Wertheim et al., 2008; Lacey et al., 2019). With regard to the animal health aspect, it was related to *S. aureus* prevalence in bovine mastitis (Magro et al., 2017). The *cna* gene, which encodes a collagen adhesion protein, was found in six MRSA strains in our study. Accordingly, Klein et al. (2012) detected a *cna* gene prevalence of 22.4% in 85 MRSA isolates of dairy cattle from Brazil. *Cna* might play a pivotal role in binding collagen in wounded, injured, or inflamed tissue, e.g., in mastitis (Madani et al., 2017). In addition, in our study, the fibronectin-binding protein encoding *fnbA* gene was present in all MRSA strains. This protein was shown to be connected to mastitis in a mouse model (Brouillette et al., 2003) and it seems to be related to more contagious *S. aureus* strains in bovine mastitis (Magro et al., 2017). The *sdrC*, *sdrD*, and *sdrE* genes, which encode the serine-aspartate repeat proteins, were found in nearly all strains in our study. In particular, the presence of the *sdrD* gene was associated with bone infections (Trad et al., 2004) and more contagious *S. aureus* strains during mastitis (Magro et al., 2017). Moreover, biofilm formation plays a crucial role in virulence of *S*. *aureus* (Costerton et al., 1999). The genes *icaA*, *icaB*, *icaC*, *icaD*, and *icaR*, which are related to biofilm formation in several staphylococcal species, were present in all MRSA isolates in our study. Since biofilm formation is of high clinical impact (Martín-López et al., 2002), this might have also been an important issue regarding animal health in the MRSA isolates in our study.

### Low Risk for Public Health

Virulence factor genes encoding the TSST, PVL, or elements of the IEC, which are associated with severe human infections, were not detected in our MRSA isolates. This is in agreement with the absence of most SaPIs and phages in the genomes, since in LA-MRSA these pathogenicity factors are often encoded in SaPIs (Ballhausen et al., 2017). SaPIbov5 and SaPIbov4, with a sequence coverage of 89–90%, were detected in 24 and 33% of our strains. Most likely, the von Willebrand factor-binding protein, a clotting factor encoded by the von Willebrand factor binding protein gene, was located on the SaPIs of the respective strains as shown by Viana et al. (2010). In particular, the SCC*mec* IVa MRSA strains from farm AA were equipped with both SaPIs. Cuny et al. (2016) associated some SCC*mec* IV and *spa*-type t011 LA-MRSA strains to human infections and Walther et al. (2018) found that 72% of ST398 and *spa*-type t011 MRSA strains in horse clinics harbored the human IEC encoded in a phiSa3 phage. Anyhow, the lack of TSST, PVL, and IEC genes in the strains from the farms investigated in our study indicates a low risk for severe human infections.

Furthermore, it has to be considered that food intoxication might appear by the consumption of raw milk or raw milk products, if SE producing MRSA strains are present in high numbers (Sergelidis and Angelidis, 2017). Yang et al. (2020) found high frequencies of SE genes in MRSA isolates of bovine mastitis cases from China. SE genes were also detected in MRSA strains of raw milk from Italy (Riva et al., 2015) and Egyptian dairy herds (El-Ashker et al., 2020). In contrast, Kadlec et al. (2019) and Kreausukon et al. (2012) did not detect any SE genes in LA-MRSA strains from German dairy farms. Accordingly, all strains investigated in our study were lacking genes for SEs, thus also lowering the possibility of a food poisoning. Moreover, the mastitis-related *S. aureus* genotype GTB, which is associated with the presence of the *sea*, *sed*, and *sej* genes (Graber et al., 2009), was not found in our study.

Our study is limited by the number of strains that were investigated with respect to the phylogenetic dynamics of MRSA strains across German dairy farms. Therefore, as a future perspective, sampling on dairy farms across Germany should be expanded and as a consequence, longitudinal core genome analysis of larger numbers of MRSA strains should be performed to better resolve the transmission pathways and evolutionary mechanisms of MRSA.

## Conclusion

The results of our study show that MRSA on German dairy farms harbor a broad repertoire of antimicrobial resistance and virulence factor genes. Some of the virulence genes are associated to mastitis, but none of them are connected to human infections. Phylogenetic analyses indicate more than 24 allelic differences of the strains across Germany with some regional spots of minor allelic diversity. Transmission of MRSA between farms may occur and MRSA strains may also be expansively transmitted within the farm environment. The prediction of antimicrobial resistance through bioinformatics tools was in agreement with the phenotypic resistance profiles. MRSA monitoring on animal farms is of high significance, since the genetic repertoire might spontaneously change due to horizontal gene transfer of MGEs and transmission pathways need to be resolved for containment of MRSA on animal farms.

## Data Availability Statement

The datasets presented in this study can be found in online repositories. The names of the repository/repositories and accession number(s) can be found below: https://www.ncbi.nlm.nih.gov/, BioProject PRJNA634452.

## Ethics Statement

Ethical review and approval was not required for the animal study because sampling of milk and nasal swabs from calves and heifers was carried out in accordance with German legislation. No ethical approval from the Institutional Ethics Committee or the National Animal Experimentation Council was required. Samples were collected by a trained veterinarian with consent from the owners of the animals. Written informed consent was obtained from the owners for the participation of their animals in this study.

## Author Contributions

TL, AS, and B-AT: study design. TL, AS, and JAH: laboratory work. TL and AS: data analysis. All authors: manuscript preparation and review.

## Conflict of Interest

The authors declare that the research was conducted in the absence of any commercial or financial relationships that could be construed as a potential conflict of interest.

## Footnotes

1 https://gitlab.com/bfr_bioinformatics/AQUAMIS/

2 https://github.com/tseemann/shovill

3 https://github.com/tseemann/mlst

4 https://cge.cbs.dtu.dk/services/

5 https://gitlab.com/bfr_bioinformatics/bakcharak

6 https://github.com/tseemann/abricate
